# Supplementary material for: Effect of moderate elevated intra-abdominal pressure on lung mechanics and histological lung injury at different positive end-expiratory pressures
Source: PLoS One. 2020 Apr 15;15(4):e0230830. doi: 10.1371/journal.pone.0230830 (PMC7159202; doi:10.1371/journal.pone.0230830)
Supplement: S1 Table — Quantitative assessment for lung injury calculated by the averaging score for five independent variables and the resulting score (from 0 = no damage to 1 = maximal alteration). (DOCX) [file pone.0230830.s002.docx]

|  | **Score per field** | | |
| --- | --- | --- | --- |
| **Parameter** | **0** | **1** | **2** |
| A: Neutrophils in the alveolar space | none | 1-5 | >5 |
| B: Neutrophils in the interstitial space | none | 1-5 | >5 |
| C: Hyaline membranes | none | 1 | >1 |
| D: Proteinaceaous debris filling the airspaces | none | 1 | >1 |
| E: Alveolar septal thickening | <2x | 2x-4x | >4x |
| Score = ((20xA) +(14xB) +(7xC) +(7xD) +(2xE)) /100 |  |  |  |

**Table 1 supplement.** The Lung Injury Scoring System. Quantitative assessment for lung injury calculated by the averaging score for five independent variables and the resulting score (from 0 = no damage to 1 = maximal alteration).
