# Supplementary material for: Effect of moderate elevated intra-abdominal pressure on lung mechanics and histological lung injury at different positive end-expiratory pressures
Source: PLoS One. 2020 Apr 15;15(4):e0230830. doi: 10.1371/journal.pone.0230830 (PMC7159202; doi:10.1371/journal.pone.0230830)
Supplement: S2 Fig — = group A with PEEP 5 cmH2O, = group B with PEEP 10 cmH2O = group C with PEEP 15 cmH2O * = p<0.05 HO vs. H6. Mean values with SEM are illustrated. (DOCX) [file pone.0230830.s004.docx]

**Figure 2 supplement.** Alterations of inspiratory transpulmonary pressure and expiratory transpulmonary pressure to an intra-abdominal pressure of 10 mmHg over 6 hours mechanical ventilation.


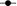
 = group A with PEEP 5 cmH_2_O.


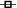
 = group B with PEEP 10 cmH_2_O.


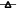
 = group C with PEEP 15 cmH_2_O.

* = p<0.05 H0 vs. H6.

Mean values with SEM are illustrated.
