# Supplementary material for: Effect of moderate elevated intra-abdominal pressure on lung mechanics and histological lung injury at different positive end-expiratory pressures
Source: PLoS One. 2020 Apr 15;15(4):e0230830. doi: 10.1371/journal.pone.0230830 (PMC7159202; doi:10.1371/journal.pone.0230830)
Supplement: S3 Fig — Mean values with SEM are illustrated. (DOCX) [file pone.0230830.s005.docx]

**Figure 3 supplement.** Wet-dry weight ratio of areas of the right lung.

Mean values with SEM are illustrated.
