# Supplementary material for: Effect of moderate elevated intra-abdominal pressure on lung mechanics and histological lung injury at different positive end-expiratory pressures
Source: PLoS One. 2020 Apr 15;15(4):e0230830. doi: 10.1371/journal.pone.0230830 (PMC7159202; doi:10.1371/journal.pone.0230830)
Supplement: Attachment — Submitted filename: Response to reviewer PLOS_one.docx [file pone.0230830.s007.docx]

Dr. Armin Kalenka

Department of Anesthesiology and Intensive Care Medicine

Hospital Bergstrasse

Viernheimer Str. 2

64646 Heppenheim

Germany

Linda L. Maerz, MD

Academic Editor

PLOS ONE

January 7th, 2020

**Point by point response to reviewers**

Dear Dr. Maerz,

First of all we would like to thank you and the two reviewers for reviewing our manuscript.

In our revised manuscript we addressed the points raised during the review process.

- The references were re-checked, re-ordered and re-numbered.
- The formatting errors were eliminated.
- The discrepancies in the years of publication in a few of the references were corrected.
- Three journals (Crit Care, ICM exp and BMC Gastro) do not provide terminal page numbers. So this item could not be corrected.
- Several errors of English language usage and a few typographical errors were corrected.

We would like to thank both reviewers for their benevolent words.

Reviewer #1:

Reviewer #1 recommend to consider another study at pressures of 15 and 20 mmHg to evaluate the optimum PEEP in this situation. We appreciate this advice. We are already done a study with IAP of 20 and 30 mmHg and will soon submit this study for publication.

Reviewer #1 also noted that clinical situations mostly not end after 6 hours like in our study. We include a new passage in our limitations to address this topic: “Clinical scenarios rarely end at 6 hours in human subjects and extending IAP and the study for a prolonged period may also reveal a difference in optimal PEEP recommendations.”

Reviewer #2:

As Reviewer #1 he noted that our study not support a decision in situations with larger increase of IAP. We therefore address this in our limitations: “Clinical scenarios rarely end at 6 hours in human subjects and extending IAP and the study for a prolonged period may also reveal a difference in optimal PEEP recommendations.”

Yours sincerely

Dr. Armin Kalenka (corresponding author)
