# Supplementary material for: Effect of moderate elevated intra-abdominal pressure on lung mechanics and histological lung injury at different positive end-expiratory pressures
Source: PLoS One. 2020 Apr 15;15(4):e0230830. doi: 10.1371/journal.pone.0230830 (PMC7159202; doi:10.1371/journal.pone.0230830)
Supplement: Attachment — Submitted filename: Response to reviewer PLOS_one_second_revision.docx [file pone.0230830.s008.docx]

Dr. Armin Kalenka

Department of Anesthesiology and Intensive Care Medicine

Hospital Bergstrasse

Viernheimer Str. 2

64646 Heppenheim

Germany

Linda L. Maerz, MD

Academic Editor

PLOS ONE

January 29^th^, 2020

**Point by point response to reviewers**

Dear Dr. Maerz,

In our second revised manuscript we addressed the points raised during the review process.

- The references were re-checked, re-ordered and re-numbered.
- Several errors of English language usage and a few typographical errors were corrected.

There were no specific points by the two reviewers which we had to address.

Yours sincerely

Dr. Armin Kalenka (corresponding author)
